# Antitumor and Anti-Metastatic Effects of Citral-Loaded Nanostructured Lipid Carrier in 4T1-Induced Breast Cancer Mouse Model

**DOI:** 10.3390/molecules25112670

**Published:** 2020-06-09

**Authors:** Noraini Nordin, Swee Keong Yeap, Heshu Sulaiman Rahman, Nur Rizi Zamberi, Nurul Elyani Mohamad, Nadiah Abu, Mas Jaffri Masarudin, Rasedee Abdullah, Noorjahan Banu Alitheen

**Affiliations:** 1 Department of Cell and Molecular Biology, Faculty of Biotechnology and Biomolecular Sciences, Universiti Putra Malaysia, Selangor 43400, Malaysia; noraininordin1303@gmail.com (N.N.); heshusr77@gmail.com (H.S.R.); rizieaq@gmail.com (N.R.Z.); elyani.mohamad@gmail.com (N.E.M.); nadiah.abu@ppukm.ukm.edu.my (N.A.); masjaffri@upm.edu.my (M.J.M.); 2 China-ASEAN College of Marine Sciences, Xiamen University Malaysia, Sepang 43900, Malaysia; skyeapxmu@gmail.com; 3 Department of Physiology, College of Medicine, University of Sulaimani, Sulaymaniyah 46001, Kurdistan Region, Iraq; 4 UKM Medical Centre, UKM Medical Molecular Biology Institute (UMBI), Cheras 56000, Kuala Lumpur, Malaysia; 5 Faculty of Veterinary Medicine, Universiti Putra Malaysia, Selangor 43400, Malaysia; rasedee@upm.edu.my; 6 Institute of Bioscience, Universiti Putra Malaysia, Selangor 43400, Malaysia

**Keywords:** citral, murine model, breast cancer, nanocarrier

## Abstract

Cancer nano-therapy has been progressing rapidly with the introduction of many novel drug delivery systems. The previous study has reported on the in vitro cytotoxicity of citral-loaded nanostructured lipid carrier (NLC-Citral) on MDA-MB-231 cells and some preliminary in vivo antitumor effects on 4T1 breast cancer cells challenged mice. However, the in vivo apoptosis induction and anti-metastatic effects of NLC-Citral have yet to be reported. In this study, the in vitro cytotoxic, anti-migration, and anti-invasion effects of NLC-Citral were tested on 4T1 breast cancer cells. In addition, the in vivo antitumor effects of oral delivery of NLC-Citral was also evaluated on BALB/c mice induced with 4T1 cells. In vitro cytotoxicity results showed that NLC-Citral and citral gave similar IC50 values on 4T1 cells. However, wound healing, migration, and invasion assays reflected better in vitro anti-metastasis potential for NLC-Citral than citral alone. Results from the in vivo study indicated that both NLC-Citral and citral have anti-tumor and anti-metastasis effects, whereby the NLC-Citral showed better efficacy than citral in all experiments. Also, the delay of tumor progression was through the suppression of the c-myc gene expression and induction of apoptosis in the tumor. In addition, the inhibition of metastasis of 4T1 cells to lung and bone marrow by the NLC-Citral and citral treatments was correlated with the downregulation of metastasis-related genes expression including MMP-9, ICAM, iNOS, and NF-kB and the angiogenesis-related proteins including G-CSF alpha, Eotaxin, bFGF, VEGF, IL-1alpha, and M-CSF in the tumor. Moreover, NLC-Citral showed greater downregulation of MMP-9, iNOS, ICAM, Eotaxin, bFGF, VEGF, and M-CSF than citral treatment in the 4T1-challenged mice, which may contribute to the better anti-metastatic effect of the encapsulated citral. This study suggests that NLC is a potential and effective delivery system for citral to target triple-negative breast cancer.

## 1. Introduction

Cancer has become one of the major public health problems in the world. A broad range of research initiatives are focused on finding a cure for cancer. According to the National Centre for Health Statistics of the USA, about 606,520 cancer mortalities were recorded in 2015. Breast cancer remains the most common type of cancer among women. [1] Although there have been drastic improvements in the cancer treatment, most of the therapies are prone to side effects and may cause cytotoxicity in the healthy cells as well. [2] Breast cancer progression is greatly linked with metastasis of the cancer cells to other organs such as lungs, liver, bone marrow, and bones. It was approximated that 85% of breast cancer patients would suffer from bone metastases. [3] Triple-negative breast cancer (TNBC), which is clinically defined by the absence of expression of estrogen receptor (ER), progesterone receptor (PR), and human epidermal growth factor receptor 2 (HER2), contributes to an approximate 15–20% of all breast cancer cases. This is one of the most aggressive subtypes of breast cancer that is associated with poor prognosis [4]. Therefore, to find a drug that not only cures cancer but also minimizes the metastasis of cancer is one of the crucial goals in cancer research. Among the breast cancer cell line, triple-negative 4T1 murine breast cancer cells have been widely used as an in vitro and in vivo model for drug discovery against TNBC as it is highly tumorigenic, metastatic, and does not express ER, PR, and HER2 receptors [5]. Previous studies have shown that citral, which is an aldehyde compound found in the essential oil from citrus fruits and lemongrass, has in vitro cytotoxicity effects on TNBC MDA-MB-231 cells [6] and in vivo antitumor effects on TNBC 4T1 cells challenged mice [7]. 

Tremendous efforts have been carried out to develop the best drug delivery system for cancer treatment by targeting the carcinoma cells without disrupting the normal cells to reduce the side effects. Cancer nanotherapy is progressing rapidly with the aim to find better ways to treat cancer. Nanostructured lipid carrier (NLC) was introduced in the late 90s to resolve the disadvantages of solid lipid nanoparticle (SLN) and is mainly claimed to improve drug solubility, enhance delivery, and sustain release of the drug-loaded. [8] As described in our published paper, the encapsulation of citral with NLC has prolonged the release and increased the solubility of citral [9]. Moreover, we have proven that the NLC-Citral is efficacious in inducing cytotoxicity on human breast cancer cell line, MDA MB-231 [9]. Oral formulation is an ideal alternative for the administration of water-soluble anticancer agent because it can increase the potency and adsorption time in cancer patients [10]. NLC has been proposed to be suitable for oral administration without causing any toxicity effects in mice [11]. In this regard, the physiochemical and stability characterization of NLC-Citral proved that NLC-Citral possessed all the characteristics for effective oral administration. However, the in vivo anti-tumor effect of NLC-Citral is uncertain as there is no such study to date. Therefore, the aim of this present study is to evaluate the anti-tumor effects of NLC-Citral on 4T1 cell-induced breast cancer in a BALB/c mice model.

## 2. Results

### 2.1. NLC-Citral Inhibited the Proliferation of 4T1 Cells In Vitro

**Figure 1:**
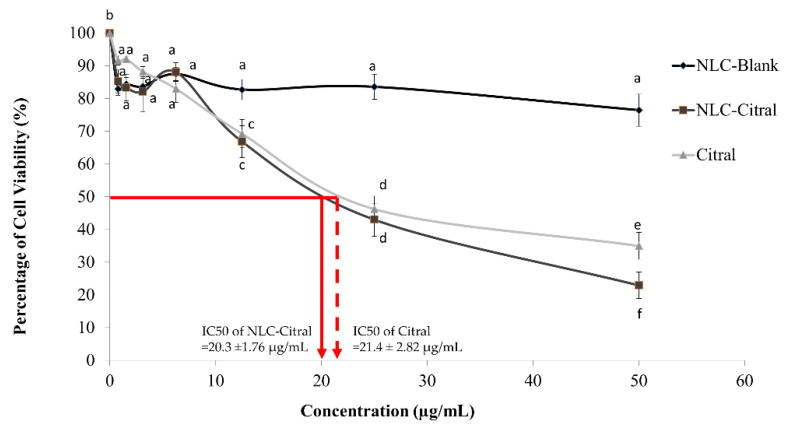
The percentage of viable 4T1 cells treated with different concentrations of NLC-Blank, NLC-Citral, and citral at 48 h in MTT assay. Each value is represented as mean ± SD and the experiment was done in triplicate. The red line indicates how the IC_50_ for NLC-Citral (full red line) and Citral (dotted red line) was obtained. Significance indicated by different alphabet was set at *p* < 0.05 comparing between groups.

Based on the MTT assay, NLC-Citral and pure citral have reduced the viability of 4T1 cells in vitro in a dose-dependent manner. According to Figure 1, the half-maximal inhibitory (IC_50_) of NLC-Citral and citral were 20.3 ±1.76 µg/mL and 21.4 ± 2.82 µg/mL respectively after 48 h of incubation whilst, for NLC-Blank the viability of the cells did not reach below 75%. 

### 2.2. NLC-Citral Reduced the Migration and Invasion of 4T1 Cells In Vitro

**Figure 2:**
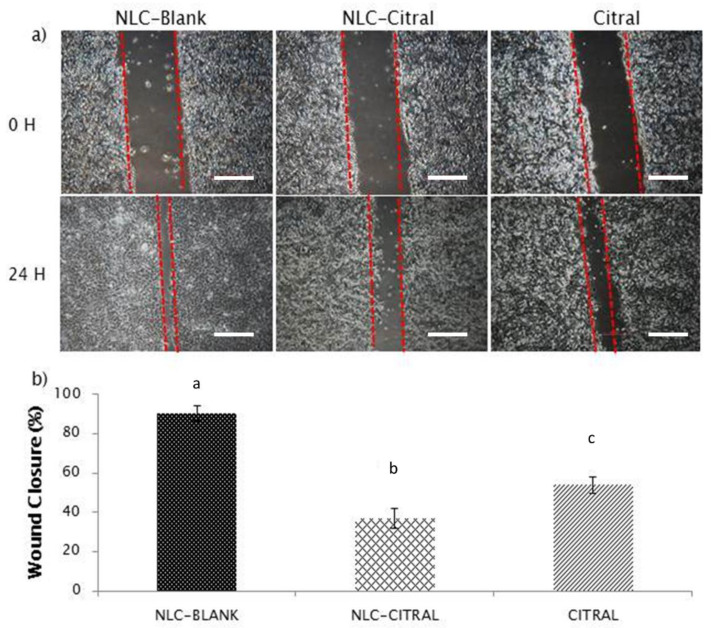
The representative images and bar chart analysis of wound healing treated with 26 µg/mL (IC40) of NLC-Citral, citral, and NLC-Blank for 24 h. The wound closure was measured in between the wound scratched. Each value in the bar chart is represented as mean ± SD and the experiment was performed in triplicate. Significance indicated by different alphabet was set at *p* < 0.05, comparing between groups. The scale bars show 500 px (13.2 cm).

**Figure 3:**
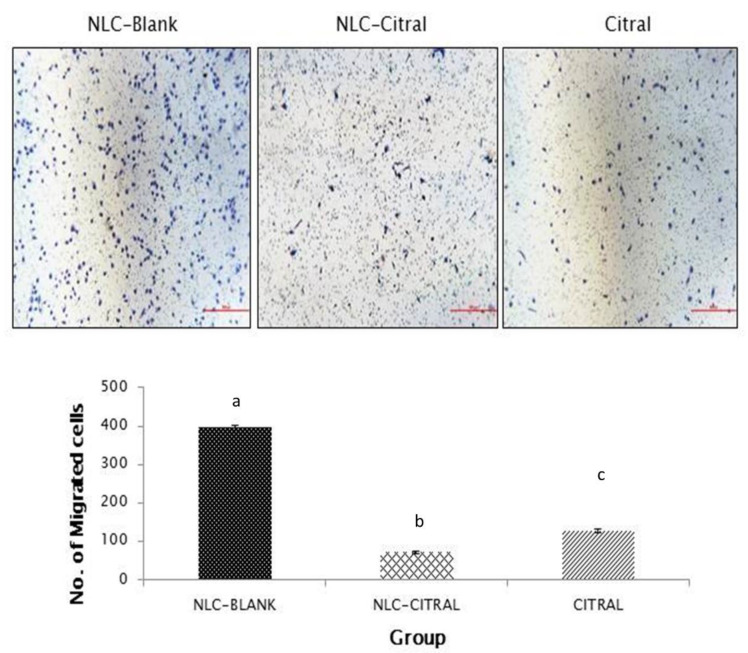
The representative images and bar chart analysis of migrated 4T1 cells through the transwell membrane after 24 h of treatment with 26 µg/mL (IC_40_) of NLC-Citral, citral, and NLC-Blank. The number of migrated cells was counted based on the presence of the blue dye stained cells. The images were viewed at 200× magnification. Each value in the bar chart is represented as mean ± SD and the experiment was performed in triplicate. Significance indicated by different alphabet was set at *p* < 0.05 comparing between groups. The scale bars show 500 px (13.2 cm).

**Figure 4:**
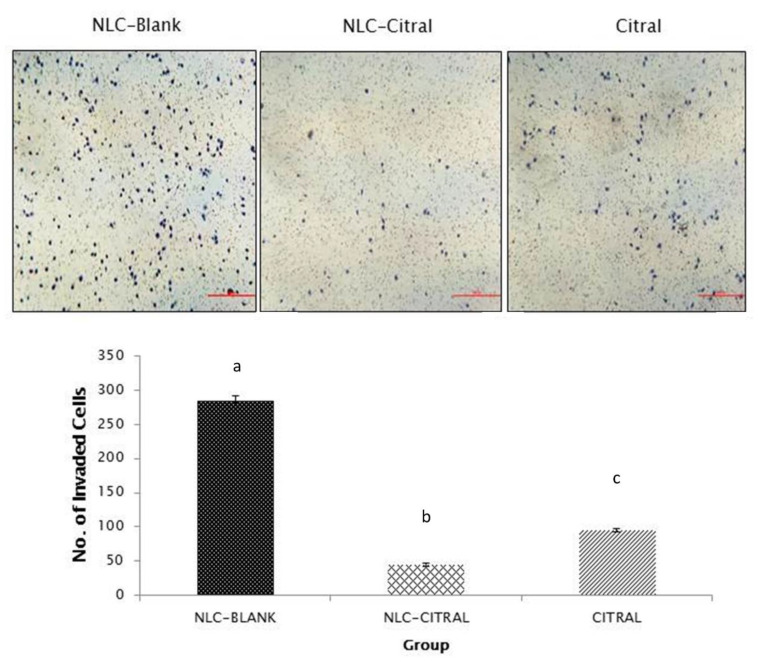
The representative images and bar chart analysis of invaded 4T1 cells through the transwell membrane after 24 h of treatment with 26 µg/mL (IC40) of NLC-Citral. The number of invaded cells was counted based on the presence of the blue dye stained cells. The images were viewed at 200× magnification. Each value in the bar chart is represented as mean ± SD, the experiment was performed in triplicate. Significance indicated by different alphabet was set at *p* < 0.05 comparing between groups. The scale bars show 500 px (13.2 cm).

To assess the anti-migration and invasion effects of the 4T1 cells treated with NLC-Citral and pure citral, in vitro wound healing assay, transwell migration, and invasion assays were employed. Figure 2 showed that the exposure of 4T1 cells to 26 µg/mL of NLC-Citral for 24 h has significantly reduced the percentage of wound closure to 36.5 ± 2.0% and 53.5 ± 2.3% than 92.6 ± 1.8% in NLC-Blank as compared to NLC-Citral and citral respectively. Additionally, in the transwell migration assay (Figure 3), NLC-Citral has significantly suppressed the migration of 4T1 cells. The number of migrated cells was significantly decreased (*p* < 0.05) by 5.5-fold in NLC-Citral-treated group compared to pure citral-treated cells with only 3-fold. Likewise, the number of invaded cells was reduced by 6-fold and 3-fold when treated with NLC-Citral and citral respectively from the invasion assay (Figure 4). Based on the results, it can be concluded that NLC encapsulation enhanced the effectiveness of citral anti-migration and anti-invasion properties on 4T1 cells. 

### 2.3. NLC-Citral Induced Apoptosis in the 4T1 Tumor

**Figure 5:**
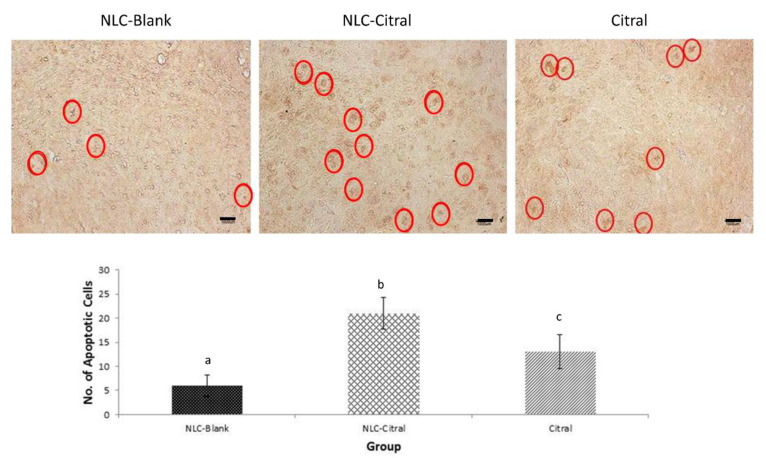
The representative images and histogram analysis of TUNEL assay of tumor from NLC-Blank, NLC-Citral, and citral groups. The red circle illustrates the DNA fragmentation detected in the tumor sections. The magnification viewed was at 100×. The scale bar shows 100 µm. Each value in the bar chart is represented as mean ± SD and the experiment was performed in triplicate. Significance indicated by different alphabet was set at *p* < 0.05 comparing between groups.

In the TUNEL assay, the apoptotic cells can be observed as dark brown color cells. Furthermore, a higher number of apoptosis cells were observed in the NLC-Citral (3.5-fold) and citral (2.1-fold) treated tumors as compared to the NLC-Blank (Figure 5). These results showed that NLC-Citral induced significant apoptosis (*p* < 0.05) of 4T1 cells in the tumors of BALB/c mice. 

### 2.4. NLC-Citral Regulated Serum Cytokines Expressions (IL-10 and IL-1β)

**Figure A1:**
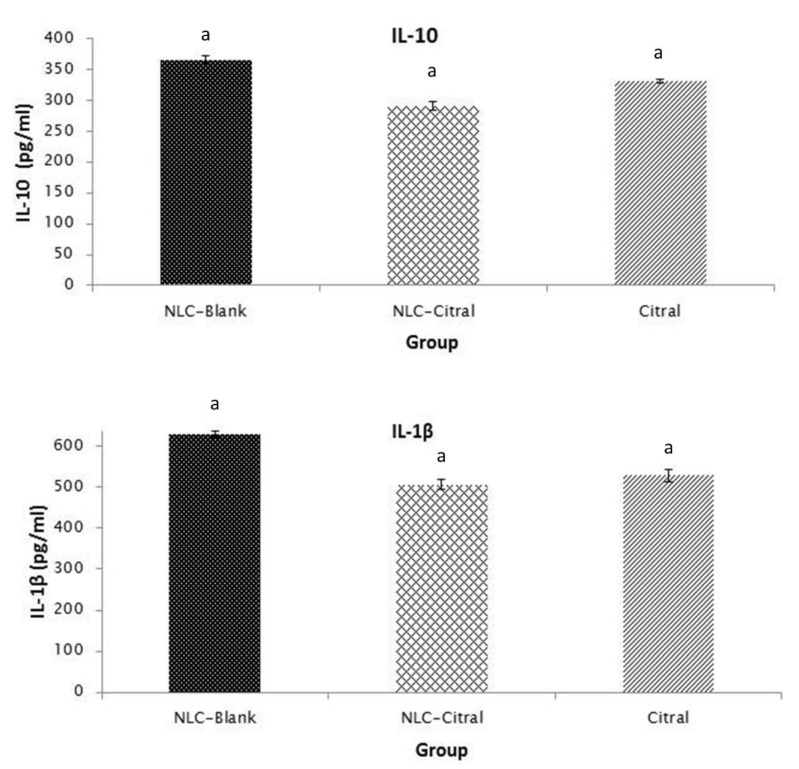
ELISA analysis of the concentration of (Upper) interleukin−10 and (Lower) interleukin-1β on blood serum of all mice. Each value in the bar charts is represented as mean ± SD, the experiment was performed in triplicate. Significance indicates by different alphabet was set at *p* < 0.05 comparing between groups.

To further confirm the anti-tumor effects of NLC-Citral on 4T1 cells, the levels of cytokines in the blood of NLC-Blank, NLC-Citral, and citral-treated mice were determined. An insignificant reduction of IL-10 level was observed in NLC-Citral-treated mice as compared to both NLC-Blank and citral-treated mice. The level of tumor pro-growth cytokine, IL-10 was reduced to 291 ± 1.5 pg/mL in NLC-Citral from 360 ± 0.8 pg/mL in NLC-Blank and 331 ± 0.40 pg/mL in citral-treated mice. Additionally, the level of IL-1β reduced from 630.3 ± 0.70 pg/mL to 506.1 ± 0.90 pg/mL in NLC-Blank and NLC-Citral respectively (Appendix A Figure A1). Nevertheless, the reduction of both IL-10 and IL-1β cytokines level was not significant in between the treatment groups. 

### 2.5. NLC-Citral Decreased Nitric Oxide and Lipid Peroxidation Levels in 4T1 Tumor

**Figure 6:**
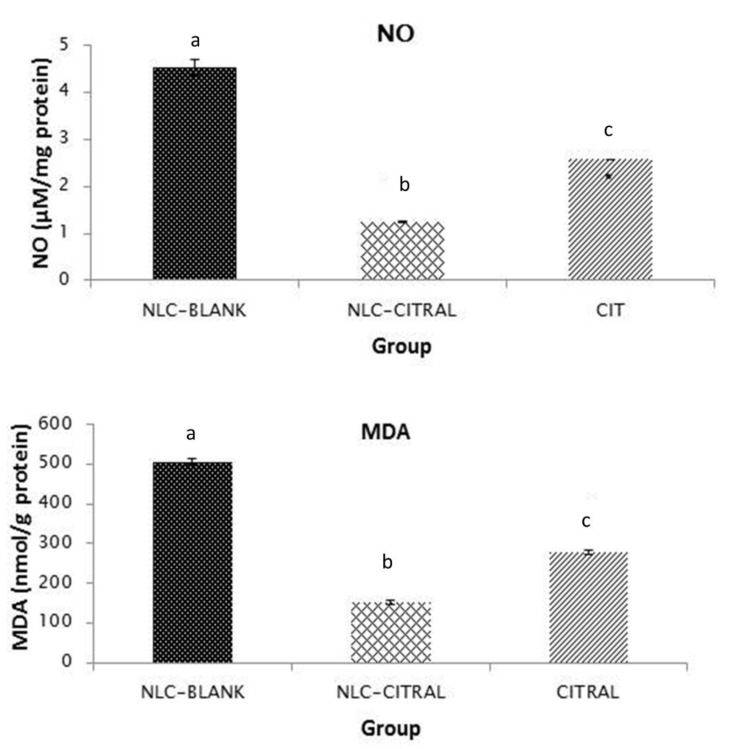
Nitric oxide (NO) and malondialdehyde (MDA) levels in the tumor in all groups. Each value in the bar charts is represented as mean ± SD, the experiment was performed in triplicates. Significance indicated by different alphabet was set at *p* < 0.05 comparing between groups.

In the in vivo analysis, the levels of lipid peroxidation of MDA and NO in the 4T1 tumor treated with NLC-Citral, citral, and NLC-Blank were measured. There was a significant reduction of the NO level in NLC-Citral (1.23 ± 0.16 µM/mg) and citral (2.5 ± 0.31 µM/mg) treated mice as compared to NLC-Blank (4.53 ± 0.86 µM/mg) (Figure 6). Similarly, the level of MDA has significantly reduced (*p* < 0.05) to 151 ± 5.45 nmol/mg in NLC-Citral from 506 ± 7.07 nmol/mg in NLC-Blank while at 278 ± 3.07 nmol/mg respectively.

### 2.6. NLC-Citral Possessed Anti-Metastatic Effects on 4T1 Cells

**Figure 7:**
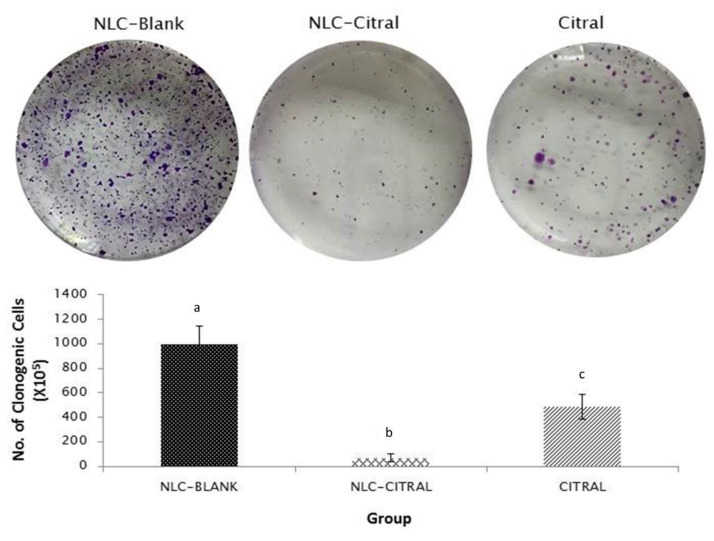
The representative images and bar chart analysis of the lung clonogenic assay at a dilution factor of 10^4^. Lung was harvested from all groups of mice. Each value in the bar chart is represented as mean ± SD, the experiment was performed in triplicate. Significance indicated by different alphabet was set at *p* < 0.05 comparing between groups.

**Figure 8:**
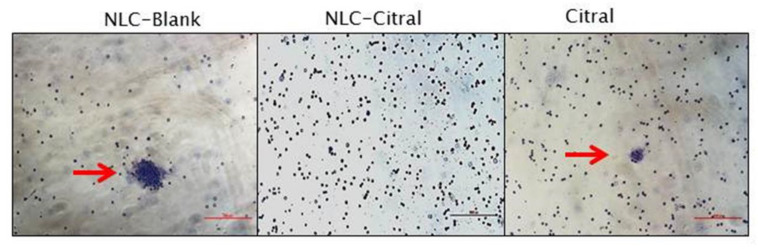
Bone marrow smear from all groups of mice. Images were viewed at 200× magnification. The red arrow indicates the 4T1 cell that has metastasized into the bone marrow.

To test the anti-metastasis effects of NLC-Citral on 4T1 cells, the clonogenic and bone marrow smearing assays were conducted. As depicted in Figure 7, the clonogenic assay showed a significant (*p* < 0.05)14-fold reduction in the numbers of 4T1 colonies metastasized to the lung of the NLC-Citral group as compared to NLC-Blank. While in the bone marrow smearing assay, the metastatic 4T1 cells were identified by the presence of abnormal clumped-cells in the NLC-Blank and citral groups while it was absent in the NLC-Citral group (Figure 8). 

### 2.7. Regulation of the Metastasis-Related Genes and Proteins Expression

**Figure 9:**
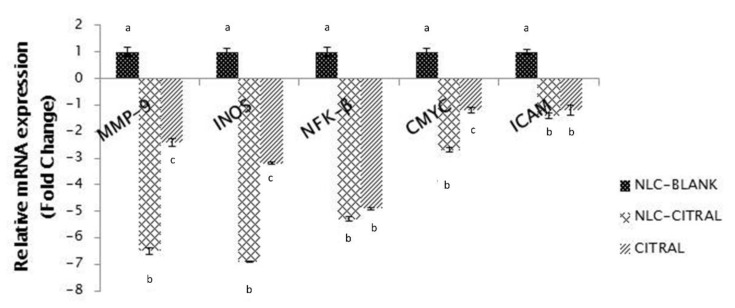
The relative mRNA expression level of MMP-9, iNOS, NFK-β, C-MYC, and ICAM. Each value is represented as mean ± SD and the experiment was performed in triplicate. Significance indicated by different alphabet was set at *p* < 0.05 comparing between groups.

**Figure 10:**
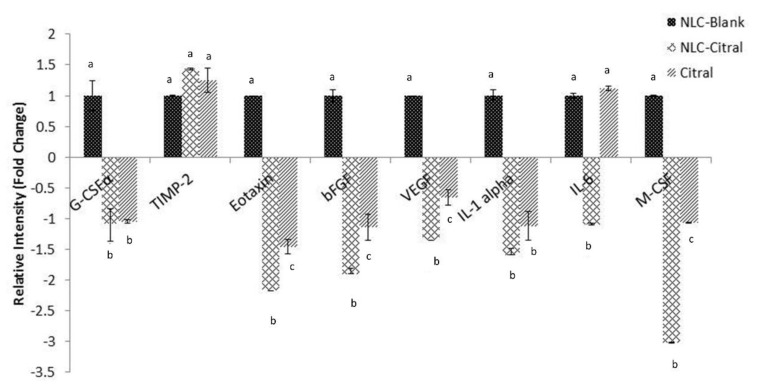
Angiogenesis proteome profiler analysis of the tumor harvested from each group. Each value is represented as mean ± SD and the experiment was performed in triplicate. Significance indicated by different alphabet was set at *p* < 0.05 comparing between groups.

To further analyze the reduction of metastasis of 4T1 cells in NLC-Citral-treated mice, the mRNA expression of five different related genes (MMP-9, iNOS, NFK-β, C-MYC, and iCAM) were determined in the excised tumor. The mRNA expression of MMP-9, iNOS, and C-MYC was down-regulated significantly in the NLC-Citral-treated mice than the mice from Citral and NLC-Blank groups (Figure 9). The expression level of MMP-9 and iNOS was observed to be down-regulated in NLC-Citral by 6.5-folds and 6.9-folds while only by 2.4-fold and 3.2-fold in citral as compared with the NLC-Blank. Also, based on the proteome profiler analysis, the level of pro-angiogenesis proteins was observed to be significantly reduced in the NLC-Citral-treated group as compared with the NLC-Blank (Figure 10). These proteins include G-CSF, macrophage colony-stimulating factor (M-CSF), TIMP-2, EOTAXIN, VEGF, bFGF, IL-1α, and IL-6. Based on the results, M-CSF protein showed the highest significant changes (*p* < 0.05) by 3.1-fold in NLC-Citral as compared with the NLC-Blank, followed by EOTAXIN (2.4-fold), and the rest of the proteins with only 1.0-fold changes in the protein expression levels. Meanwhile, most of the protein expression levels were not showing any significant difference when compared to citral except for M-CSF and EOTAXIN proteins.

## 3. Discussion

In our previous study, the potential anti-tumor effect of NLC-Citral was ascertained using MDA-MB-231 cells in vitro [12]. In this study, the oral efficacy of NLC-Citral as compared to the citral in suppressing the breast tumor growth was tested in 4T1-challenged mice treated for 28 days. In the previous study, we have shown that the size and weight of the 4T1-tumors in the NLC-Citral-treated mice were substantially reduced compared to the NLC-Blank and the citral groups reflected that NLC-Citral was more effective compared to citral alone. [12] In addition, both NLC-Citral and citral-treated mice were not observed with any signs and symptoms of toxicity (results not shown). Reduction of tumor size may be contributed by the induction of apoptosis by citral, particularly NLC-Citral, which was recorded with higher events of TUNEL positive cells. TUNEL assay was mainly used to assess the DNA damage in the cancer cells, which indicates that the cells were undergoing apoptosis [13]. This result has shown that NLC encapsulation enhanced the oral bio-efficacy of citral. This effect is similar to a recent study, which claimed that NLC encapsulation enhances the oral bio-efficacy of Baicalin in Wistar rats [14]. Based on our qPCR analysis, c-myc expression was differentially regulated in the NLC-Citral and citral-treated 4T1 cells. C-MYC is a key regulator gene for cell growth and the deregulation of this gene is associated with breast cancer development [15]. Over-expression of C-MYC as a primary oncogene result in the increment of cancer cell proliferation and blocking its metabolic pathway would lead to a new approach in cancer treatment [16]. Therefore, the reduction of C-MYC expression level could inhibit tumor growth proliferation, which subsequently promotes the complete elimination of cancer cells. 

In this study, NLC-Citral has successfully reduced the levels of MDA and NO in NLC-Citral-treated mice. NO is a signaling molecule that regulates several cellular processes such as immune response, apoptosis, and angiogenesis [17]. A high level of NO has been proposed to mediate the metastatic process and tumor growth progression in breast and cervical cancers [18]. 

NF-κB is a crucial player in inflammation and cancer development in which its up-regulation indicates the aggressiveness of breast cancer tumors and extreme tendency to metastasize [19]. Interestingly, the NLC-Citral treatment group was observed with the reduction of the NF-κB gene expression in the mice breast tumor. NF-κB activation increases the expression of important downstream cellular signaling molecules in the pathway such as iNOS as an inflammatory mediator in cancer [20].

Consequently, the mRNA expression level of iNOS was down-regulated in the NLC-Citral group as well. Induction of iNOS is correlated with inflammation and functions as a tumor growth promoter [21]. The observed decrease of NO in the NLC-Citral-treated mice was associated with the down-regulation of the iNOS gene. Similarly, the report has claimed that NLC-ZER substantially inhibited the lipid peroxidation, pro-inflammatory cytokines, and inflammatory mediators in the 4T1-challenged mice [22]. Therefore, the reduction of genes as a major player in an inflammation-related process is pivotal in breast cancer treatment and chemotherapy.

In the cancer setting, tumor growth depends on the angiogenic sprout from the existing vasculature [23]. In this study, significantly less 4T1 cells have migrated and invaded in the NLC-Citral-treated as shown in the wound healing, migration, and invasion assays. Additionally, there was a significant reduction of 4T1 cells metastasized in the lung as well as no abnormal cells were found in the bone marrow of the NLC-Citral-treated mice suggesting that NLC-Citral could effectively inhibit the metastatic ability of the 4T1 cells to the lung and bone marrow. It has been reported that anti-angiogenic agents reduced the metastasized breast cancer primary tumor to the lung and lymph nodes [24].

To further evaluate the anti-metastasis properties of the NLC-Citral on breast cancer, the level of metastasis-related cytokines including IL-1α, IL-1β, IL-6, and IL-10 were quantified. These cytokines are also known for its contribution to the angiogenic and metastasis of various cancers such as breast, melanoma, lung, and prostate [25]. Based on the results from proteome profiler, NLC-Citral reduced the level of IL-6 and IL-1α expression in the 4T1 tumor. More specifically, IL-6 and IL-1α cytokines are known to enhance the promotion of tumor growth, angiogenesis, and metastasis of aggressive human cancers such as breast cancer [26]. On the other hand, serum level of IL-1β and IL-10, which are also metastasis-related cytokines were quantified by ELISA. However, the serum level of IL-1β and IL-10 was slightly reduced but non-significant compared to the untreated group. These results indicate that NLC-Citral and citral suppressed the metastasis by targeting the IL-1β and IL-6 expression in the tumor without sufficient regulation of circulating IL-1α and IL-10 cytokines. 

On the other hand, NF-κB has been reported to be involved in the expression of pro-metastasis and pro-angiogenic genes such as MMP-9 and VEGF. [27] Based on the qPCR analysis in the NLC-Citral group, MMP-9 was significantly down-regulated as compared to citral and control groups. MMP-9 degrades the extracellular matrix of cells and caused tissue disruption for the invasion of tumor cells in the metastasis process [28]. Other studies have shown that the expression of MMP-9 is significantly higher in the metastatic breast cancer than non-metastatic patients [29]. In addition, various tumor growth factor are crucial for the migration and development of breast cancer such as VEGF [30]. It was found that the VEGF and Eotaxin levels were higher in the bone microenvironment because of the breast cancer colonization in the femurs of the mice [31]. These findings are in accordance with the results obtained in this study where NLC-Citral reduced the level of VEGF and eotaxin while increased the TIMP-2 protein expression in the treated mice. Furthermore, M-CSF was identified to produce angiogenic factors such as VEGF and MMPs [32]. In this study, the level of M-CSF in the NLC-Citral-treated group was significantly lower than the citral and control. The other growth factors that were found to be lowered in the NLC-Citral-treated mice were G-CSF and b-FGF. The recent study has reported that G-CSF drives tumor cell proliferation and metastasis in human xenograft and murine neuroblastoma [33]. Meanwhile, a clinical study has reported that highly expressed bFGF significantly mediated the metastasis of lung cancer to lymph nodes [34]. Therefore, the inhibition of inflammation and angiogenesis-related markers by NLC-Citral verified the potential of NLC-Citral as an anti-metastatic agent for breast cancer therapeutics. Based on this study, NLC-Citral managed to inhibit the proliferation, motility, and invasiveness of 4T1 cells in vitro and significantly induced apoptosis-inhibited inflammation and impedes metastasis of 4T1 cells in a murine model.

## 4. Materials and Methods 

### 4.1. Preparation and Characterization of NLC-Citral 

Citral was loaded into NLC by high-pressure homogenization technique using hydrogenated palm oil (HPO), lipoid S-100 (Merck Millipore, Germany), olive oil (Basso, Italy), thimerosal, D-Sorbitol, Tween-80, and citral 95%. Characterization of NLC-Citral was determined using transmission electron microscope (TEM), zeta potential, drug loading, and drug release studies. TEM and zeta sizer analyses revealed that NLC-Citral is a nano-size particle with an average diameter size of 54.12 ± 0.30 nm. Meanwhile, the zeta potential of NLC-Citral was −12.73 ± 0.34 mV with an entrapment efficiency of 98.9 ± 0.124%, and drug loading was 9.84 ± 0.041%. The details of the preparation and characterization of the NLC-Citral was reported in Nordin et al. [9]. 

### 4.2. Cell Culture Condition 

4T1 murine breast cancer cell line was purchased from American Type Culture Collection (ATCC) (ATCC, Rockville, MD, USA). The cells were maintained in a complete growing media (RPMI-1640) (Sigma-Aldrich, St. Louis, MO, USA) supplemented with 10% fetal bovine serum (FBS) (GIBCO, USA), and 1% penicillin-streptomycin antibiotic (GIBCO, Gaithersburg, MD, USA). All cells were grown in culture flasks kept at 37 °C and 5% CO_2_ [22].

### 4.3. MTT Assay 

The in vitro colorimetric cytotoxicity effect of NLC-Citral on 4T1cells was done through the MTT assay according to the method described earlier [35]. Briefly, cells were harvested, counted, and seeded at 1 × 10^4^ cells per well in a 96-wells plate for 48 h before being treated with the serial diluted NLC-citral or Citral at 50, 25, 12.5, 6.25, 3.12, 1.56, and 0.78 µg/mL. The following day, cells were treated with various concentrations of the sample and incubated for 48 h. After 48 h, 20 µL of (5 mg/mL) MTT (Merck, USA) reagent was added to each well and incubated again for 3 h. Next, the solution was removed and 100 µL of dimethyl sulfoxide (DMSO) was added to the wells. Afterward, the plate was read at 575 nm by using the microtiter plate reader µQuant (Bio-Tek Instrument, Winooski, VT, USA). The results were analyzed as the percentage of the proliferation of the cells with respect to the concentration of the samples treated. IC50 was obtained by interpolating the IC50 of the y-axis with the dosage at the x-axis of dose-response curves.

### 4.4. In Vitro Scratch Assay 

To assess the anti-migration effect of NLC-Citral toward 4T1 cells, the in vitro scratch assay was administered [36]. Sixteen µg/mL (IC_40_) of NLC-Citral and citral were used in the treatment of cancer cells and the cells were plated to create 80% confluent monolayer overnight. Later on, the cell monolayer in a straight line scraped with a sterile p200 pipette tip to create scratches. Subsequently, the media was replaced with the treated media. Cells were then incubated for 24 h. The image of wound closure was captured at 0, 12, and 24 h of incubation time (Nikon, Tokyo, Japan). 

### 4.5. In Vitro Transwell Migration and Invasion Assay 

To further evaluate the anti-metastasis effect of NLC-Citral on 4T1 cells, the in vitro transwell migration and invasion assays were conducted [37]. For invasion assay, the cell culture insert membrane (SPL, Korea) was coated with 650 µL of diluted matrigel and incubated for 3 h to solidify the gel. The coating gel was prepared by diluting the matrigel to a ratio of 1: 2 with serum-free media. Meanwhile, for the migration assay, the membrane was not coated with anything. Next, 4 × 10^5^ cells per well were seeded on top of the solidified membrane. Next, in the bottom compartment of the chamber, 2 mL of supplemented media with 16 µg/mL of NLC-citral and citral were added. Then, the cells were incubated for 24 h in a 37 °C incubator. After that, the non-migrated/invaded cells at the top part of the membrane were scraped away with the cotton swab. The lower part of the membrane was fixed with 1 mL methanol for 30 min and preceded with 0.5% crystal violet for 30 min. The pictures of the migrated/invaded cells through the membrane were viewed and photographed for further analysis (Nikon, Japan). This assay was performed in triplicates. 

### 4.6. Animals 

Female adult BALB/c mice aged 6 to 8 weeks were purchased from the Animal House of Faculty of Veterinary Medicine, Universiti Putra Malaysia, Serdang, Selangor (UPM, Serdang, Malaysia). The mice were acclimatized for 1 week and housed under the standard condition at 24 °C ± 1 °C under 12-h dark and light cycle. The mice were provided pellet and water *ad libitum* during the whole period of study. This study was approved by the Animal Care and Use Committee, Faculty of Veterinary Medicine, Universiti Putra Malaysia (UPM/IACUC/AUP-R098/2014). 

### 4.7. Animals Grouping and Treatment 

The mice were grouped into three groups (*n* = 6 for each group): (1) NLC-Blank group, (2) NLC-Citral-treated group, and (3) Citral-treated group. Mice in all three groups were inoculated with 1 × 10^5^ 4T1 cells in RPMI-1640 media via subcutaneous injection [38]. All treated mice were fed orally with NLC-Citral and citral with 50 mg/kg daily using oral gavage for 28 days starting on the first day of inoculation. The dose was selected based on the studies conducted previously [12]. Whereas, the negative control group was treated with the vehicle was used to encapsulate the citral (NLC-Blank). Samples were collected from five mice and each sample was subjected to three technical replicates for the following experiments. 

### 4.8. TUNEL Assay 

This assay was used to determine the apoptosis in the tumor inoculated tissues using the Dead End^TM^ TUNEL system kit (Promega, Fitchburg, WI, USA) according to the manufacturer’s protocol. In brief, the tissue sections mounted on the glass slides were deparaffinized in xylene for 5 min twice. The slides then were rehydrated in different concentrations of ethanol for 5 min each subsequently. Next, the slides were immersed in PBS for 10 min. Next, tissues were permeabilized with Proteinase K for 30 min and then fixed with paraformaldehyde for 15 min. The tissues mounted on the slides were equilibrated using an equilibrium buffer before being labeled with terminal deoxynucleotidyl transferase (TdT). After that, the slides were blocked with hydrogen peroxide. Then the slides were incubated in streptavidin-horseradish peroxidase (HRP) and developed using 3,3’-diaminobenzidine (DAB) substrate before being mounted in glycerol. Lastly, the slides were viewed under the light microscope (Motic, Hong Kong). Apoptosis scoring of the tumor which is stained dark brown was counted from at least five different fields in stained tumor tissue slides.

### 4.9. Nitric Oxide Detection Assay 

The level of NO in the tumor harvested from all groups was measured according to the protocol provided in the Griess reagent kit (Sigma, St. Louis, MO, USA). Tumors were meshed with the plunger and filtered with cell strainer in PBS. Then, the supernatants were mixed with the Griess reagent and incubated for 30 min before being measured at 548 nm using a microplate reader (Beckman Coulter, Miami, FL, USA).

### 4.10. Malondialdehyde Detection 

The MDA content is an indicator of lipid peroxidation in the tumors. The level of lipid peroxidation from all groups was assessed by thiobarbituric acid (TBA) reactive substances [39]. Briefly, the harvested tumor cells were mashed and filtered with the cell strainer in PBS before centrifuging at 2000× for 5 min. About 200 µL of supernatant was mixed with PBS, butylated hydroxytoluene (BHT), and trichloroacetic acid (TCAA), followed by incubation on ice for 2 h. After that, the mixtures were centrifuged at 2000× *g* and mixed with thiobarbituric acid (TBA) and EDTA before they were heated to 95 °C for 15 min. Next, the mixture was chilled, and the absorbance was read at 532 nm using a spectrophotometer (Beckman Coulter, USA). The standard curve was prepared using tetra methoxy propane (Sigma-Aldrich, USA) and the MDA concentration was calculated.

### 4.11. Serum Cytokine Detection 

Cytokine analysis from the serum of untreated and treated mice was performed using the Duoset ELISA IL-10 and IL-1β assay kits (R&D Systems, Minneapolis, MN, USA) according to the manufacturer’s protocol. First, the 96-well plates were coated with captured-antibody and incubated overnight at 4 °C. Next, the plate was washed thrice with wash buffer and blocked with blocking buffer for 1 h at room temperature. The plate was then washed twice and incubated with standard dilutions and samples for 2 h. After that, the plate was added with the detection antibody and incubated for 2 h. Next, streptavidin-HRP was added to the plate and incubated for 20 min before the substrate was added to the plate. The reaction was stopped with the addition of stop buffer when the resulting color was developed. The plate was then read at 450 nm and 570 nm using a microplate reader (Bio-Tek Instrument, Winooski, VT, USA). The levels of IL-10 and IL-1β in the sample were measured based on the standard curve of the standard solution. 

### 4.12. Clonogenic Assay

On the day of sacrifice, the lung was harvested from the untreated and treated groups before washing with PBS. The harvested lung was cut into small pieces, digested with 2 mg/mL collagenase type IV (BD, USA), and incubated for 1 h in a 37 °C water bath. Next, the lung was mashed and filtered through the cell strainer. Then, it was centrifuged at 500× *g* for 5 min and washed twice with PBS. The pellets were collected and resuspended in RPMI media with 6-thioguanine for clonogenic growth. The suspension cells were plated in 6-well plates by serial dilutions and kept in the incubator at 37 °C and 5% CO2 for 10 days. After that, the media was removed, and the plate was fixed with methanol for 30 min. The plates were stained with 0.5% crystal violet for 2 h before rinsing with distilled water and air-dried. The stained cells were counted in each of the plates for each group [40].

### 4.13. Bone Marrow Smear 

Femurs from the untreated and treated mice were collected for the bone marrow (BM) collection. The BM was washed with PBS using the syringe and collected in a petri dish. Next, the BM solution was smeared on the glass slides and air-dried. Then, the slides were fixed with methanol for 30 min and stained with Giemsa stain and incubated for 2 h at room temperature [41]. Finally, the slides were viewed under the inverted light microscope (Motic, Hong Kong).

### 4.14. Quantitative Real-Time PCR Assay of Metastatic Related Genes for In Vivo Tumor

Tumor tissues from the mice were harvested and placed in RNAlater (Ambion, Austin, TX, USA) overnight at 4 °C before being minced and transferred into −80 °C. Then, the tumors were snap cooled in liquid nitrogen and meshed before the total RNA was extracted using the Qiagen RNAeasy Mini Kit (Qiagen, Hilden, NRW, Germany) and quantified using nano-spectrophotometer (Beckton Coulter, USA). The purity was assured to be within the ratio of 1.8–2.0 at A260/A280. Then, the total RNA was converted into cDNA using the Maxima First Strand cDNA synthesis kit (Thermo Scientific, Waltham, MA, USA) and was run into a thermal cycler (Labnet, Edison, NJ, USA). Next, the qPCR assay was run using SYBR Select Master Mix (Life Technologies, California, CA, USA) in the Eco Illumina (Illumina, San Diego, CA, USA) to quantify the expression of the selected genes (Table 1). The qPCR reaction was set at 95 °C for 10 min, 95 °C of denaturation for 40 cycles, and 55–60 °C of annealing/extension for 15–30 s. The quantity of the target and housekeeping genes (GAPDH and ACTB) were calculated according to a standard curve and were measured by Eco study software (Illumina, USA). The expression levels of NLC-Citral and citral was compared to the NLC-Blank [42].

### 4.15. Proteome Profiler Mouse Angiogenesis and Metastasis Protein for In Vivo Tumor

The protein expression was analyzed using the Mouse Angiogenesis Antibody Array kit (RayBiotech, Peachtree Corners, GA, USA). The tumors were immersed in liquid nitrogen and mashed before being lysed with RIPA buffer supplemented with the pre-made protease inhibitor cocktail tablet (ROCHE, Basel, Switzerland). The lysed proteins were extracted using QIAshredder (Qiagen, Germany) and the concentrations were measured by Bradford assay (Sigma, USA). On the other hand, the antibody array membrane was transferred into the incubation tray, then blocked with blocking buffer for 30 min. Samples were then incubated with the antibody array overnight at 4 °C. Next, the tray was washed thrice with wash buffer before being incubated with each Biotinylated Antibody Cocktail and Streptavidin-HRP for 2 h. After that, the tray was washed again and air-dried. The membrane was scanned using the Axon Gene Pix 4000 B (Molecular Devices, San Jose, CA, USA) after the detection buffer was added. The data were extracted using Axon Gene Pix Pro 6.1 software (Molecular Devices, USA) and analyzed. 

### 4.16. Statistical Analysis

All experiments were done in triplicate and the average values were obtained. All of the results were expressed as the means and standard deviations of all mice (*n* = 6) for each group. The statistical analysis was performed using GraphPad Prism (Version 6.0). Statistical significance (*p* < 0.05) was assayed by a one-way ANOVA analysis with Tukey’s posthoc. The comparison of the statistical analyses was determined between the NLC-Citral and citral-treated groups to the NLC-Blank as a negative control group.

## 5. Conclusions

In conclusion, NLC encapsulation enhanced the anti-tumor effect of citral through induction of apoptosis and suppression of cancer cell metastasis in TNBC 4T1-challenged mice. These results suggest that NLC is a promising nanocarrier for anti-tumor natural products particularly citral. This work documents for the first time that citral formulated into the NLC system is a promising candidate for breast cancer therapeutics.

## Figures and Tables

**Table 1:** List of the gene name, accession number, and sequence of the primers used in the real-time qPCR analysis.

Gene Name	Accession Number	Sequence of Primers
Mmp-9	NM_013599.3	F: 5′-GCCGACTTTTGTGGTCTTCC-3′ R: 5′-GGTACAAGTATGCCTCTGCCA-3′
iNOS	NM_010927.3	F: 5′-GCACCGAGATTGGAGTTC-3′ R: 5′-GAGCACAGCCACATTGAT-3′
Nf-κb	NM_0086892	F: 5′- CCTGCTTCTGGAGGGTGATG -3′ R: 5′- GCCGCTATATGCAGAGGTGT -3′
C-myc	NM_010849.4	F: 5′-TGATGTGGTGTCTTGGAGAA-3′ R: 5′-CGTAGTTGTGCTGGTGAGTG-3′
Icam1	NM_010493.2	F: 5′- TGCTCAGGTATCCATCCATCC-3′ R: 5′- ACGGTGCCACAGTTCTCAA-3′
GAPDH	NM_008084.3	F: 5′-GAAGGTGGTGAAGCAGGCATC-3′ R: 5′-GAAGGTGGAAGAGTGGGAGTT-3′
ACTB	NM_007393.3	F: 5′-TTCCAGCCTTCCTTCTTG-3′ R: 5′- GGAGCCAGAGCAGTAATC-3′
